# Leaf Shape Responds to Temperature but Not CO_2_ in *Acer rubrum*

**DOI:** 10.1371/journal.pone.0049559

**Published:** 2012-11-12

**Authors:** Dana L. Royer

**Affiliations:** Department of Earth and Environmental Sciences, Wesleyan University, Middletown, Connecticut, United States of America; University College London, United Kingdom

## Abstract

The degree of leaf dissection and the presence of leaf teeth, along with tooth size and abundance, inversely correlate with mean annual temperature (MAT) across many plant communities. These relationships form the core of several methods for reconstructing MAT from fossils, yet the direct selection of temperature on tooth morphology has not been demonstrated experimentally. It is also not known if atmospheric CO_2_ concentration affects leaf shape, limiting confidence in ancient climate reconstructions because CO_2_ has varied widely on geologic timescales. Here I report the results of growing *Acer rubrum* (red maple) in growth cabinets at contrasting temperature and CO_2_ conditions. The CO_2_ treatment imparted no significant differences in leaf size and shape, while plants grown at cooler temperatures tended to have more teeth and more highly dissected leaves. These results provide direct evidence for the selection of temperature on leaf shape in one species, and support a key link in many leaf-climate methods. More broadly, these results increase confidence for using leaf shape in fossils to reconstruct paleoclimate.

## Introduction

Leaf size and shape commonly covary with climate [1], [2], and paleobotanists have long used this covariation to reconstruct paleoclimate from fossil floras [2]–[4]. Little et al. [5] recently compiled 351 publications that investigate the response of leaf size and shape to climate in extant and fossil vegetation, underscoring their widespread study. In particular, leaf teeth have been recognized for nearly a century as correlating with mean annual temperature (MAT) [2]–[4], [6], [7]. The proportion of woody, non-monocotyledonous species at a site with toothed leaf margins correlates inversely with MAT in most regions of the world [2], [4], [7]–[9]. Tooth size, tooth number, and degree of leaf dissection also covary inversely with MAT [4], [10], [11]. In short, plants in cold climates are more likely to have toothed leaf margins, and their teeth are larger and more numerous. These morphological traits have been used in univariate and multivariate models for estimating MAT from fossil floras [2], [4], [7], [12]–[14].

In all current leaf-climate models for reconstructing MAT, tooth-related variables contribute the most explanatory power [2], [4]. Despite the seeming importance of leaf teeth, how can one be confident that they are causally-related to MAT and not simply correlated secondarily? One helpful but indirect approach to this problem is to investigate whether leaf teeth are more adaptive in colder climates. Based on measurements of photosynthesis and transpiration, Royer and Wilf [15] concluded that many teeth exhibit a pulse in gas exchange during the first few weeks of the growing season (see also [16]); this should boost sap flow rates, increasing the delivery of nutrients to young, expanding leaves [17]. The early-season pulse is most pronounced in colder climates and is absent in untoothed leaves [15]. This function of leaf teeth may be increasingly adaptive in colder climates where the potential for growth is more limiting; in other words, leaf teeth help plants ramp up to maximum carbon production rates sooner in the growing season than an equivalent plant with no teeth. In warmer climates, the water cost associated with teeth may outweigh any benefits for maximizing the growing season length [15], [18].

While these gas-exchange patterns are compelling for drawing a causal link between MAT and leaf shape, there are alternative functional explanations for leaf teeth. These include herbivore avoidance [19], [20] (but see [21]), mechanical support associated with leaf thickness [22]–[24], a deciduous canopy [16], [24], and the release of excess root pressure [25]. Given the array of possible selective factors on leaf teeth, it is difficult to isolate the degree of causality of any one factor. The best approach is to maximize variation in one factor (e.g., MAT) while minimizing variation in other potential factors. In this spirit, Royer et al. [11] noted that tooth abundance correlated strongly with MAT within four broadly-distributed species (*Acer rubrum*, *Prunus serotina*, *Ostrya virginiana*, *Carpinus caroliniana*) at 16 U.S. east coast sites. In a follow-up study with *Acer rubrum* (red maple), most aspects of tooth size, tooth number, and degree of leaf dissection correlated significantly with MAT at 77 U.S. east coast sites, matching global site-mean patterns [26]. A common garden experiment with red maple, where seeds from two seed sources (Ontario, Canada and Florida, USA) were each grown at two sites with contrasting MAT (Rhode Island, USA and Florida, USA), revealed that both growth site and seed source affected leaf shape; plants grown in the colder Rhode Island garden or sourced from the colder Ontario seed bank produced more highly dissected leaves with more teeth [27].

These studies demonstrate that MAT likely affects tooth-related variables in *Acer rubrum*. However, even with the common garden experiment, herbivory, leaf thickness, and other climatic variables were not fully controlled for or reported. A major goal of this study is to test directly the effect of growth temperature on leaf shape in *Acer rubrum* in a more controlled setting using growth cabinets. These results, in turn, can provide a fuller context for the application of leaf-climate methods in the fossil record.

In addition to the factors just discussed, it is possible that atmospheric CO_2_ concentration impacts leaf shape [2], [28]. Changes in atmospheric CO_2_ affect the carbon economy in most plants, most critically through an increase in dry matter production and a reduction in stomatal conductance [29]. If leaf teeth also affect plant carbon economy (e.g., via a boost in gas exchange early in the growing season), then it is possible that CO_2_ influences tooth-related variables. Because atmospheric CO_2_ has varied greatly on geologic timescales [30], it is important to understand its role in leaf-climate methods.

**Table 1:** Statistical evaluation of the impact of temperature and CO_2_ on leaf size and shape in *Acer rubrum*.

	Temperature			CO_2_		
Variable	Low	High	*P*	Low	High	*P*
**Tooth abundance**						
Number of teeth	77.6 (7.2)	48.6 (4.5)	0.01	53.8 (4.1)	45.3 (4.6)	0.18
Number of teeth/internal perimeter (cm^−1^)	2.82 (0.28)	1.98 (0.11)	0.05†	2.48 (0.27)	2.06 (0.27)	0.29
Number of teeth/leaf area (cm^−2^)	3.25 (0.50)	2.63 (0.23)	0.28	3.46 (0.65)	2.84 (0.51)	0.47
**Leaf dissection**						
Perimeter ratio	1.27 (0.02)	1.18 (0.02)	0.04	1.23 (0.02)	1.20 (0.03)	0.35
Circularity	0.26 (0.02)	0.30 (0.02)	0.16	0.31 (0.02)	0.33 (0.02)	0.56
Compactness	49.3 (3.1)	43.5 (3.3)	0.22	42.3 (2.6)	40.5 (3.0)	0.66
**Leaf and tooth size**						
Leaf area (cm^2^)	27.6 (2.8)	20.8 (3.1)	0.12	20.7 (2.5)	21.9 (2.5)	0.73
Leaf perimeter (cm)	36.0 (2.1)	29.3 (2.6)	0.06	28.5 (2.0)	28.4 (1.6)	0.96
Tooth area (cm^2^)	1.59 (0.17)	1.37 (0.29)	0.51†	1.42 (0.17)	1.28 (0.16)	0.54
Average area of a single tooth (cm^2^)	0.030 (0.004)	0.034 (0.006)	0.59	0.036 (0.004)	0.036 (0.005)	0.91
Tooth area/internal perimeter (cm)	0.055 (0.004)	0.050 (0.008)	0.59†	0.059 (0.004)	0.050 (0.004)	0.18
Tooth area/leaf area	0.059 (0.004)	0.060 (0.007)	0.97	0.071 (0.004)	0.059 (0.005)	0.06

Note.– Variables are grouped by relatedness to tooth abundance, leaf dissection, or leaf and tooth size. Values in parentheses are the standard error of the mean. *P*-values are based on t-tests with a Dunn-Šidák correction to account for multiple comparisons; for pairwise comparisons that failed Levene’s test for equality of variances, t-tests assuming non-homogeneous variance were used (denoted by †). Internal perimeter is the leaf perimeter after the removal of teeth; perimeter ratio is the leaf perimeter divided by the internal perimeter; circularity is 4×π×(leaf area)/(leaf perimeter)^2^; compactness is (leaf perimeter)^2^/(leaf area). Variables without stated units are unitless.

Many studies have investigated the effect of CO_2_ on leaf area (e.g., [31]), but very few have examined tooth-related variables. Thomas and Bazzaz [32] observed an increase in leaf dissection (perimeter-area allometry) at high CO_2_ in *Taraxacum officinale* (dandelion). However, dandelion is heteroblastic, and leaf carbohydrate level is commonly linked to heteroblastic leaf development (see [32]). Thus, it is unclear if these results are applicable to non-heteroblastic species. In a second study, Gregory [31] found little effect of CO_2_ on a suite of 29 leaf size and shape characters in *Quercus alba* (white oak). However, Gregory’s results may not be applicable to some leaf-climate methods because the characters were defined categorically and cannot be readily translated to tooth size and number (e.g., “teeth regular”, “teeth acute”, “teeth compound”). A second goal of the current study is to test directly the effect of CO_2_ on leaf shape variables related to tooth size, tooth number, and leaf dissection in *Acer rubrum*.

## Materials and Methods

Seeds from a single *Acer rubrum* L. branch were collected in May 2011 from a street tree in Middletown, Connecticut, USA (Middletown MAT = 9.6°C; mean annual precipitation = 1170 mm [33]). The branch was gently shaken to release its seeds. No specific permits were required for sampling because the tree is on public land and did not involve endangered or protected species. Within hours, seeds were wrapped in wet paper towel, sealed in a single plastic bag, and stored in a refrigerator for six weeks. This process of cold stratification increases germination yields in *Acer rubrum* [34].

**Figure 1:**
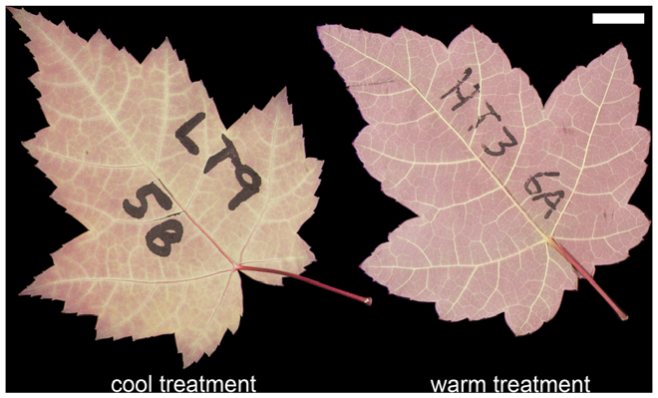
Representative leaves of *Acer rubrum* from the cool (left) and warm (right) temperature treatments. Both leaves are similar in area and match their treatment means for tooth number and perimeter ratio (leaf perimeter divided by perimeter after removal of teeth). Scale bar = 1 cm.

**Figure 2:**
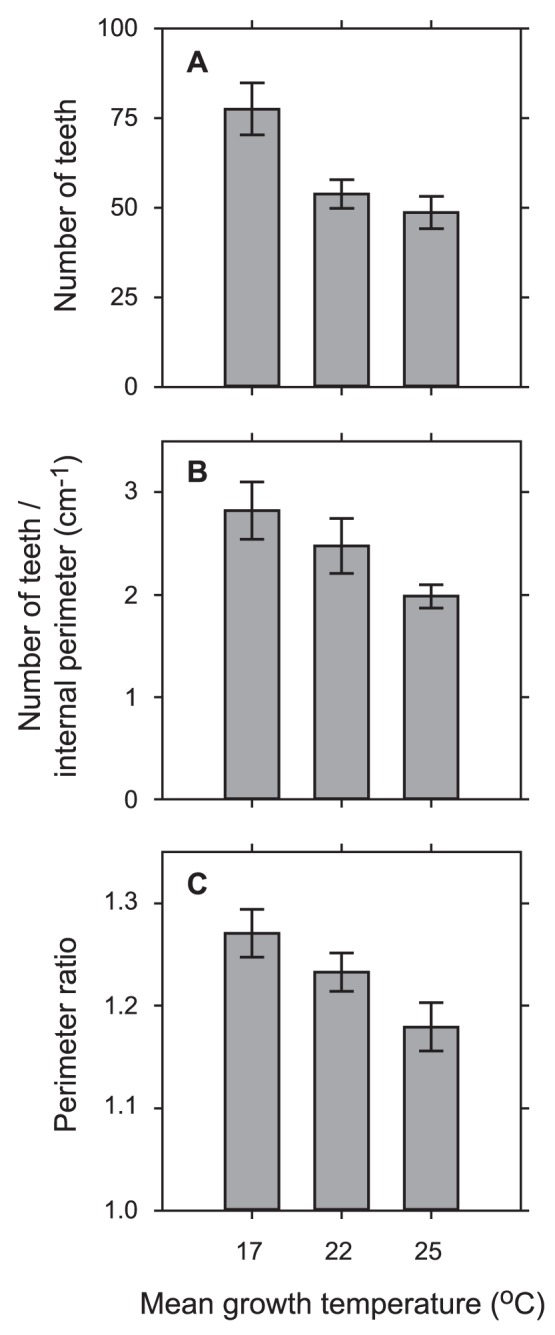
Comparisons from temperature experiment for three leaf shape variables. Data from the low CO_2_ treatment of the CO_2_ experiment are also included (middle bars in panels) because the environmental conditions for these leaves were identical to that in the temperature experiment but with an intermediate growth temperature (see Materials and Methods). Standard errors of the mean are plotted. All pairwise comparisons from the temperature experiment are significantly different (*P*≤0.05; see Table 1). Internal perimeter is the leaf perimeter after the removal of teeth; perimeter ratio is the leaf perimeter divided by the internal perimeter.

The CO_2_ experiment was carried out first, followed by the temperature experiment twelve weeks later. In both experiments, each of 48 seeds were pressed into moist potting soil (Pro-Max BX, Premier Horticulture, Quakertown, Pennsylvania, USA) in a 0.33 L pot fertilized with slow-release granules (All-purpose flower and vegetable continuous release plant food, Scotts, Marysville, Ohio, USA). Pots were randomly divided into two groups and each group placed into an independently-controlled growth cabinet (Conviron E7/2; Winnipeg, Manitoba, Canada). In both experiments, relative humidity was fixed at 70% and the day/night cycle at 16/8 hours. In the CO_2_ experiment, the CO_2_ treatments were 500 and 1000 ppm; day/night temperatures were fixed at 16°C/25°C (mean temperature = 22°C). In the temperature experiments, the day/night temperature treatments were 11°C/20°C (mean temperature = 17°C) and 19°C/28°C (mean temperature = 25°C); CO_2_ was fixed at 500 ppm.

Plants were watered regularly to maintain soil moisture; excess water passing through the pots was discarded. Plants were rotated between cabinets on a weekly basis to minimize cabinet effects. After seven weeks, the ten most vigorous plants were transplanted into 1.4 L pots with fresh potting soil and fertilizer.

Leaves were harvested after 12–15 weeks, depending on plant growth rate. At their time of harvest, plants had produced six to ten sets of leaves (leaves in red maple are oppositely arranged). Leaves were immediately photographed (Nikon Coolpix 995 camera, Nikon, Melville, New York, USA); all digital images are available from the author upon request. The procedure for analyzing the sizes and shapes of leaves is discussed fully by Royer et al. [11] and Huff et al. [10]. Briefly, in Photoshop (Adobe Systems, San Jose, California, USA) minor defects along the leaf margin are corrected using the line tool and petioles are separated from the leaf blade. Teeth are then separated from the leaf blade; most teeth are bounded by two sinuses, but see Royer et al. [11] and Huff et al. [10] for exceptions. Once the leaf is prepared, its size and shape is quantified with ImageJ (http://rsbweb.nih.gov/ij/) [35]. All measured variables are related to tooth abundance, degree of leaf dissection, or tooth and leaf size; see Table 1 for a complete list, along with definitions for the less commonly-known variables. For the temperature experiment, leaf dry thickness was also measured using calipers (Mitutoyo dial caliper, Mitutoyo, Kanagawa, Japan); for each leaf, two measurements were made near the base, avoiding first-order veins.

Four leaves per plant were analyzed; the unit of replication for all statistical tests is the plant (*n* = 10 per treatment). Ontological effects were minimized by analyzing, in most plants, the sixth and seventh leaf pairs. *P*-values are based on t-tests with a Dunn-Šidák correction to account for multiple comparisons; t-tests assuming non-homogeneous variance were used for pairwise comparisons that failed Levene’s test for equality of variances.

## Results

Leaf teeth were more abundant in plants grown in cooler temperatures (Table 1; Figures 1 and 2). Leaves from the colder temperature treatment also tended to be larger in size and perimeter, although these differences were not significant (Table 1). Nonetheless, the temperature effect on tooth abundance became non-significant when normalized for leaf area, but remained significant when normalized for leaf perimeter (Table 1; Figure 2).

Leaves grown at lower temperature also tended to be more highly dissected. A significant difference was observed in perimeter ratio, which is leaf perimeter divided by internal perimeter (perimeter after removal of teeth) (Table 1; Figure 2). Low temperature leaves also had higher perimeter/area ratios, expressed as circularity (4×π×[leaf area]/[leaf perimeter]^2^) and compactness ([leaf perimeter]^2^/[leaf area]), but these differences were not statistically significant (Table 1). No significant temperature effects were observed for leaf thickness (*P* = 0.66) or aspects of tooth size (Table 1).

In contrast to the temperature experiment, no significant differences in leaf size or shape were discerned in the CO_2_ experiment (Table 1).

## Discussion

Red maples grown in cooler temperatures produce leaves that are more highly dissected and have more teeth. These data arguably provide the strongest case to date for a direct effect of growth temperature on the morphology of leaf teeth. This is because all other known factors that influence tooth development were fully accounted for (e.g., herbivory, leaf thickness, water availability; see Introduction and [24]). Additionally, atmospheric CO_2_ had no discernible impact on any size or shape trait.

Compared with the red maple common garden experiment of Royer et al. [27], fewer significant temperature effects were observed here. Most importantly, in the common garden experiment plants grown in a cooler climate (or sourced from a seed bank with a colder climate) produced leaves with a higher perimeter/area ratio (calculated as circularity and compactness) and with more teeth per unit leaf area. Similar trends were detected in the current study, but the treatment differences were not significant (Table 1). One difference in study design was the length of treatment exposure: 27 months in the common garden experiment (three leaf flushes) versus three months here (one leaf flush). A longer treatment spanning multiple leaf flushes might result in clearer treatment differences, but would require much larger growth cabinets. Alternatively, in the common garden experiment other factors that influence leaf shape may have been present and not properly accounted for.

### Concluding Remarks

The temperature and CO_2_ experiments presented here provide strong support for the use of leaf-climate methods for at least three reasons. First, the link between temperature and leaf shape is likely primary (causal). Second, the potential interaction with atmospheric CO_2_ appears minimal (see also [31]); this is good news for fossil applications because constraints on paleo-CO_2_ are often uncertain [30]. Third, both this study and the common garden experiment [27] establish that leaf shape responds quickly and plastically to temperature in *Acer rubrum*; selection on genetic drift is not necessary. If this phenotypic plasticity is common in other taxa, then it increases the likelihood that fossil reconstructions of temperature are robust, even during times of rapid climate change.

## References

[1] Halloy SRP, Mark AF (1996) Comparative leaf morphology spectra of plant communities in New Zealand, the Andes and the European Alps. Journal of the Royal Society of New Zealand 26: 41–78.

[2] Wolfe JA (1993) A method of obtaining climatic parameters from leaf assemblages. US Geological Survey Bulletin 2040: 1–71.

[3] Bailey IW, Sinnott EW (1915) A botanical index of Cretaceous and Tertiary climates. Science 41: 831–834. 17835989 10.1126/science.41.1066.831

[4] Peppe DJ, Royer DL, Cariglino B, Oliver SY, Newman S, et al (2011) Sensitivity of leaf size and shape to climate: global patterns and paleoclimatic applications. New Phytologist 190: 724–739. 21294735 10.1111/j.1469-8137.2010.03615.x

[5] Little SA, Kembel SW, Wilf P (2010) Paleotemperature proxies from leaf fossils reinterpreted in light of evolutionary history. PLoS ONE 5 (12): e15161. 10.1371/journal.pone.0015161 PMC3008682 21203554

[6] Bailey IW, Sinnott EW (1916) The climatic distribution of certain types of angiosperm leaves. American Journal of Botany 3: 24–39.

[7] Wolfe JA (1979) Temperature parameters of humid to mesic forests of Eastern Asia and relation to forests of other regions of the Northern Hemisphere and Australasia. US Geological Survey Professional Paper 1106: 1–37.

[8] Greenwood DR (2005) Leaf form and the reconstruction of past climates. New Phytologist 166: 355–357. 15819898 10.1111/j.1469-8137.2005.01380.x

[9] Wilf P (1997) When are leaves good thermometers? A new case for Leaf Margin Analysis. Paleobiology 23: 373–390.

[10] Huff PM, Wilf P, Azumah EJ (2003) Digital future for paleoclimate estimation from fossil leaves? Preliminary results. Palaios 18: 266–274.

[11] Royer DL, Wilf P, Janesko DA, Kowalski EA, Dilcher DL (2005) Correlations of climate and plant ecology to leaf size and shape: potential proxies for the fossil record. American Journal of Botany 92: 1141–1151. 21646136 10.3732/ajb.92.7.1141

[12] Wing SL, Greenwood DR (1993) Fossils and fossil climate: the case for equable continental interiors in the Eocene. Philosophical Transactions of the Royal Society London B 341: 243–252.

[13] Stranks L, England P (1997) The use of a resemblance function in the measurement of climatic parameters from the physiognomy of woody dicotyledons. Palaeogeography Palaeoclimatology Palaeoecology 131: 15–28.

[14] Gregory-Wodzicki KM (2000) Relationships between leaf morphology and climate, Bolivia: implications for estimating paleoclimate from fossil floras. Paleobiology 26: 668–688.

[15] Royer DL, Wilf P (2006) Why do toothed leaves correlate with cold climates? Gas exchange at leaf margins provides new insights into a classic paleotemperature proxy. International Journal of Plant Sciences 167: 11–18.

[16] Baker-Brosh KF, Peet RK (1997) The ecological significance of lobed and toothed leaves in temperate forest trees. Ecology 78: 1250–1255.

[17] Cramer MD, Hawkins H-J, Verboom GA (2009) The importance of nutritional regulation of plant water flux. Oecologia 161: 15–24. 19449035 10.1007/s00442-009-1364-3

[18] Wing SL, Bao H, Koch PL (2000) An early Eocene cool period? Evidence for continental cooling during the warmest part of the Cenozoic. In: Huber BT, MacLeod KG, Wing SL, editors. Warm climates in earth history. Cambridge: Cambridge University Press. 197–237.

[19] Brown VK, Lawton JH (1991) Herbivory and the evolution of leaf size and shape. Philosophical Transactions of the Royal Society London B 333: 265–272.

[20] Lamont A (1970) Problems of leaf ecology. Scottish Journal of Science 1: 104–143.

[21] Rivero-Lynch AP, Brown VK, Lawton JH (1996) The impact of leaf shape on the feeding preference of insect herbivores: experimental and field studies with *Capsella* and *Phyllotreta* . Philosophical Transactions of the Royal Society London B 351: 1671–1677.

[22] Givnish TJ (1978) Ecological aspects of plant morphology: leaf form in relation to environment. Acta Biotheoretica (Supplement: Folia Biotheoretica No 7) 27: 83–142.

[23] Givnish TJ (1979) On the adaptive significance of leaf form. In: Solbrig OT, Jain S, Johnson GB, Raven PH, editors. Topics in plant population biology. New York: Columbia University Press. 375–407.

[24] Royer DL, Peppe DJ, Wheeler EA, Niinemets Ü (2012) Roles of temperature and life-history traits in controlling toothed vs. untoothed leaf margins. American Journal of Botany 99: 915–922. 22494908 10.3732/ajb.1100428

[25] Feild TS, Sage TL, Czerniak C, Iles WJD (2005) Hydathodal leaf teeth of *Chloranthus japonicus* (Chloranthaceae) prevent guttation-induced flooding of the mesophyll. Plant, Cell and Environment 28: 1179–1190.

[26] Royer DL, McElwain JC, Adams JM, Wilf P (2008) Sensitivity of leaf size and shape to climate within *Acer rubrum* and *Quercus kelloggii* . New Phytologist 179: 808–817. 18507771 10.1111/j.1469-8137.2008.02496.x

[27] Royer DL, Meyerson LA, Robertson KM, Adams JM (2009) Phenotypic plasticity of leaf shape along a temperature gradient in *Acer rubrum* . PLoS ONE 4 (10): e7653. 10.1371/journal.pone.0007653 PMC2764093 19893620

[28] Beerling DJ (1998) The future as the key to the past for palaeobotany? Trends in Ecology and Evolution 13: 311–316. 21238319 10.1016/s0169-5347(98)01334-2

[29] Long SP, Ainsworth EA, Rogers A, Ort DR (2004) Rising atmospheric carbon dioxide: plants FACE the future. Annual Review of Plant Biology 55: 591–628. 10.1146/annurev.arplant.55.031903.141610 15377233

[30] Royer DL (2006) CO_2_-forced climate thresholds during the Phanerozoic. Geochimica et Cosmochimica Acta 70: 5665–5675.

[31] Gregory KM (1996) Are paleoclimate estimates biased by foliar physiognomic responses to increased atmospheric CO_2_? Palaeogeography Palaeoclimatology Palaeoecology 124: 39–51.

[32] Thomas S, Bazzaz F (1996) Elevated CO_2_ and leaf shape: are dandelions getting toothier? American Journal of Botany 83: 106–111.

[33] Hijmans RJ, Cameron SE, Parra JL, Jones PG, Jarvis A (2005) Very high resolution interpolated climate surfaces for global land areas. International Journal of Climatology 25: 1965–1978.

[34] Peroni PA (1995) Field and laboratory investigations of seed dormancy in red maple (*Acer rubrum* L.) from the North Carolina piedmont. Forest Science 41: 378–386.

[35] Abramoff MD, Magelhaes PJ, Ram SJ (2004) Image processing with ImageJ. Biophotonics International 11: 36–42.

